# Magnetic nanoparticles in primary neural cell cultures are mainly taken up by microglia

**DOI:** 10.1186/1471-2202-13-32

**Published:** 2012-03-22

**Authors:** Josephine Pinkernelle, Pilar Calatayud, Gerado F Goya, Hisham Fansa, Gerburg Keilhoff

**Affiliations:** 1 Institute of Biochemistry and Cell Biology, Otto-von-Guericke University Magdeburg, Leipziger Str. 44, 39120 Magdeburg, Germany; 2 Instituto de Nanociencia de Aragón (INA), University of Zaragoza, Mariano Esquillor s/n, 50018 Zaragoza, Spain; 3 Departamento de Física de la Materia Condensada, Facultad de Ciencias, Universidad de Zaragoza, 50009 Zaragoza, Spain; 4 Department of Plastic, Reconstructive and Aesthetic Surgery, Hand Surgery, Klinikum Bielefeld, Teutoburger Str. 50, 33604 Bielefeld, Germany

**Keywords:** Magnetic nanoparticles, Primary neuronal cells, Microglia, Organotypic spinal cord

## Abstract

**Background:**

Magnetic nanoparticles (MNPs) offer a large range of applications in life sciences. Applications in neurosciences are one focus of interest. Unfortunately, not all groups have access to nanoparticles or the possibility to develop and produce them for their applications. Hence, they have to focus on commercially available particles. Little is known about the uptake of nanoparticles in primary cells. Previously studies mostly reported cellular uptake in cell lines. Here we present a systematic study on the uptake of magnetic nanoparticles (MNPs) by primary cells of the nervous system.

**Results:**

We assessed the internalization in different cell types with confocal and electron microscopy. The analysis confirmed the uptake of MNPs in the cells, probably with endocytotic mechanisms. Furthermore, we compared the uptake in PC12 cells, a rat pheochromocytoma cell line, which is often used as a neuronal cell model, with primary neuronal cells. It was found that the percentage of PC12 cells loaded with MNPs was significantly higher than for neurons. Uptake studies in primary mixed neuronal/glial cultures revealed predominant uptake of MNPs by microglia and an increase in their number. The number of astroglia and oligodendroglia which incorporated MNPs was lower and stable. Primary mixed Schwann cell/fibroblast cultures showed similar MNP uptake of both cell types, but the Schwann cell number decreased after MNP incubation. Organotypic co-cultures of spinal cord slices and peripheral nerve grafts resembled the results of the dispersed primary cell cultures.

**Conclusions:**

The commercial MNPs used activated microglial phagocytosis in both disperse and organotypic culture systems. It can be assumed that *in vivo *application would induce immune system reactivity, too. Because of this, their usefulness for *in vivo *neuroscientific implementations can be questioned. Future studies will need to overcome this issue with the use of cell-specific targeting strategies. Additionally, we found that PC12 cells took up significantly more MNPs than primary neurons. This difference indicates that PC12 cells are not a suitable model for natural neuronal uptake of nanoparticles and qualify previous results in PC12 cells.

## Background

Nanoparticles have recently received increased attention in the life sciences. Because of their small size, their cellular uptake mechanisms such as endocytosis [1,2] and the possibility to functionalize them with biofunctional active groups, nanoparticles offer a large range of applications [3-5]. They can be tagged with cell-specific target sites [6,7] and if they are magnetic, they can be moved and influenced by an external magnetic field [8]. One example of these features is cellular hyperthermia of tumours, a novel clinical protocol in which the MNPs are heated up by an alternating magnetic field and tumour cells are destroyed by thermal energy [9].

Another interesting application of magnetic nanoparticles is magnetofection™. This technique uses them as a carrier for nucleic acids and enhances cell transfection with an external magnetic field [10]. MNPs are already routinely used for contrast enhancement in magnetic resonance imaging, drug delivery, magnetic cell separation, and tissue repair studies [3,11].

One idea for an application in neuroscience is the use of MNPs to promote regeneration within the peripheral (PNS) and central nervous system (CNS). The outcome after injury repair in the nervous system is often poor, even in the PNS [12,13]. Here, different cell types participate in the regeneration of the injured nerve. After a peripheral nerve lesion, the axon distal from the injury site degenerates and Schwann cells and later macrophages clean the neural tubes of cell debris and myelin, the so-called Wallerian degeneration. Additionally, Schwann cells produce growth factors and form bands of Büngner as guiding paths for the regrowing axons, building a regeneration-promoting environment [14].

In the CNS, regeneration of injured axons is even more difficult. Unlike Schwann cells, oligodendroglia do not show phagocytic activity. Therefore, an inhibitory environment for axonal regeneration is evident and the clearance of the injury site of cell and myelin debris is much slower than in the PNS. Additionally, the regeneration-promoting environment produced by the Schwann cells in the PNS is absent [15].

Nanoparticles now offer the possibility to influence such processes locally. Mittnacht et al. [16] for example delivered RhoA-specific siRNA by nanoparticles into PC12 cells as a neuronal cell model. Thereby, they suppressed the signal transduction pathway for inhibitory proteins, resulting in enhanced neurite outgrowth of the PC12 cells after stimulation with nerve growth factor (NGF). In contrast, Hamasaki et al. [17] used magnetically labelled neural progenitor cells, which were located by an external magnetic field, to promote axon growth in organotypic co-cultures of brain cortex and spinal cord.

Although there is a trend to use self-produced nanoparticles, reproducibility among different studies would be higher if not every study was based on distinct particles. Thus, we used commercially available MNPs and analyzed their uptake in cells of the nervous system. Unfortunately, there is little literature concerning primary cell cultures and nanoparticles, especially with a neuroscientific background. Mostly, previous studies used different kinds of cell lines such as PC12 as a neuronal model [16,18], human umbilical vein endothelial cells (HUVECs) [19] and Infinity™ telomerase-immortalised primary human fibroblasts (hTERT-BJ1) [4,20]. However, cell lines are often tumour cells or immortalised cells, behaving differently than primary cells [21,22].

For this reason, we compared neuronal differentiated PC12 cells with primary neuronal cells according to their uptake of green fluorescent MNPs. Primary neuronal cells were used from rat mixed neuronal/glial cerebellar cell cultures. These neuronal cells are mainly granular neurons cultured in a more *in vivo *like environment having cell contacts with different kinds of glial cells occurring in the cerebellum. Thus, we analyzed the uptake of MNPs for the different kinds of glial cells, too. Together with the analysis of rat primary cell cultures of the PNS, mixed Schwann cell/fibroblast cultures, both culture systems give a good idea which cells will take up nanoparticles *in vivo *for a future therapeutic approach.

To verify our results in the dispersed cell cultures, organotypic co-cultures of spinal cord slices and peripheral nerve grafts as a more complex model were additionally analyzed. The organotypic co-cultures mimic the tissue architecture of both parts of the nervous system and allow screening of the cellular uptake of MNPs in a more tissue-like environment.

## Methods

### Magnetic nanoparticles

Commercial green fluorescent MNPs produced by Chemicell (4415 nano-screenMAG-ARA, Berlin, Germany) were used. These MNPs have a magnetite core covered by a lipophilic green fluorescent dye and a polysaccharide matrix of glucuronic acid, a derivate of glucose, for additional functionalization. Because of the carboxyl group of the coating polymer, the particles become anionic in solution. Zeta potential measurements of these MNPs suspended in cell culture media revealed a negative charge on the particles; this finding was also observed in the presence of serum supplement. MNPs were diluted the day before use with three different serum-supplemented media: For primary cell cultures MNPs were diluted in Dulbecco's modified Eagle medium (DMEM) medium with 10% fetal calf serum (FCS), 6 g/l D-glucose and 1% penicillin/streptomycin (pen/strep), resulting in a hydrodynamic MNP diameter of 190 nm (± 2 nm) and a zeta potential of -6.1 mV. PC12 cells were cultured with MNPs diluted in Roswell Park Memorial Institute (RPMI) medium with 10% FCS, 50 ng/ml NGF (Sigma, St. Louis, USA), 2 mM L-glutamine and 1% pen/strep, yielding a hydrodynamic MNP diameter of 199 nm (± 1 nm) and a zeta potential of -40.0 mV. For the organotypic co-cultures, MNPs were diluted in 50% Eagle's minimal essential medium (MEM), 25% Hank's balanced salt solution (HBSS), 25% FCS, 33.3 mM D-glucose, 1% pen/strep and 100 ng/ml recombinant rat glia cell line-derived neurotrophic factor (GDNF, R&D systems, Minneapolis, USA) giving a hydrodynamic diameter of 185 nm (± 1 nm) and a zeta potential of -12.8 mV.

### Animal care

All animal experiments were carried out in accordance with the guidelines of the German Animal Welfare Act. The study was approved by the Animal Care and Use Committees of Saxony-Anhalt. Formal approval to conduct the experiments described was obtained from the animal subjects review board of our institution and can be provided upon request. All efforts were made to minimize the number of animals used and their suffering.

### Cerebellar cultures

5-8 day neonatal rats were decapitated and the cerebellum was separated. Meninges were removed in serum-free DMEM containing 6 g/l D-glucose and 1% pen/strep. Subsequently, the cerebelli were mechanically dissociated (18 and 23 gauge needles) in serum-free DMEM and centrifuged at 1500 rpm for 5 min. Cells were resuspended in serum-supplemented DMEM with 10% FCS, 6 g/l D-glucose, 1% pen/strep, counted and seeded for immunocytochemistry in 12-well plates with 1*10^6 ^cells per well on poly-D-lysine-coated coverslips (18 or 15 mm). For viability assay, cerebellar cells were seeded in poly-D-lysine-coated Petri dishes (35 mm) with a cell density of 2.5*10^5 ^cells per dish.

Cells were incubated at 37°C in a humidified 6% CO_2 _atmosphere and medium was changed 24 h after preparation to remove cell debris.

For the uptake quantification based on immunocytochemistry, the medium was replaced with MNP medium (50 μg/ml MNPs) at different time points depending on cell type and different proliferation patterns of the various cell types. For example, the number of neurons decreases over the first week in cerebellar cultures [23,24] and oligodendroglia differentiate from precursor cells after more than one week [25-27]. Quantification of our mixed cerebellar cultures respective the culture composition revealed that they are composed of 45.5% (± 16.7%) astroglia, 32.1% (± 19.2%) microglia, 24.3% (± 14.6%) neurons and no oligodendroglia on day *in vitro *(DIV) 5. On DIV 7, they contain 35.2% (± 17.3%) microglia, 32.5% (± 17.2%) astroglia, 13.7% (± 10.4%) neurons and 2.1% (± 3.4%) oligodendroglia. On DIV 13, they are composed of 48.4% (± 28.4%) microglia, 38.3% (± 24.8%) astroglia, 1.5% (± 2.8%) neurons and 3.0% (± 4.1%) oligodendroglia.

To quantify the uptake of neurons, MNP medium was added on DIV 4. MNP uptake in astroglia and microglia was analyzed on DIV 7 and in oligodendroglia on DIV 13. Simultaneously, MNP-free medium was added to control cells.

For viability assay 10, 50 or 100 μg/ml MNP-containing medium was added to the cultures on DIV 6. At the same time, MNP-free medium was added to control cells. Cells were incubated for 24 h, washed with phosphate buffered saline (PBS) and used further for immunocytochemistry or for viability assay.

### Schwann cell/fibroblast cultures

5-7 day old rats were decapitated, the spinal ganglia were taken out and collected in a solution of 2 ml serum-free DMEM (6 g/l D-glucose, 1% pen/strep), 40 μl collagenase (0.05%), 100 μl hyaluronidase (0.1%) and 2 ml dispase II (1.25 U/ml). In this enzyme-supplemented medium the isolated spinal ganglia were incubated at 37°C in a 6% CO_2 _humidified atmosphere for ca. 3.5 h to dissociate the cells enzymatically. The cells were additionally dissociated mechanically using injection needles (18 and 23 gauge needles) and centrifuged at 1500 rpm for 5 min. Afterwards the cells were resuspended in serum-supplemented medium (DMEM, 10% FCS, 6 g/l D-glucose, 1% pen/strep).

For immunocytochemistry, 18 or 15 mm coverslips in 12-well plates were coated with laminin (0.05 mg/ml) before seeding 5*10^4 ^cells per well.

For viability assay cells were seeded on laminin-coated Petri dishes (35 mm) with a cell density of 2.5*10^5 ^cells per dish.

Cells were incubated at 37°C in a humidified 6% CO_2 _atmosphere and the medium was changed 24 h after preparation to remove cell debris. Culture composition was quantified and revealed 62.7% (± 17.0%) Schwann cells and 37.3% (± 17.0%) fibroblasts on DIV 7. For immunocytochemistry and viability assay, the medium was replaced with MNP medium on DIV 6. MNP medium contained 50 μg/ml MNPs for immunocytochemistry and 10, 50 and 100 μg/ml MNPs for the viability assay. MNP-free medium was added to control cells. After 24 h, cells were washed with PBS and used further.

### Cell line

PC12 cells were seeded on poly-D-lysine coated culture flasks and cultured with RPMI medium supplemented with 10% FCS, 2 mM L-glutamine and pen/strep. After 24 h, cells were differentiated with 50 ng/ml NGF for 6 days. Differentiated cells were seeded afterwards in 12-well plates with 1.25*10^5 ^cells per well on poly-D-lysine coated coverslips and kept in differentiation medium for 3 days before the medium was replaced with differentiation medium supplemented with 50 μg/ml MNPs. After 24 h, cells were washed with PBS and fixed for immunocytochemistry.

### Organotypic spinal cord co-cultures

Organotypic spinal cords co-cultured with peripheral nerve grafts were prepared according to Vyas et al. [28] with slight modifications. For the cultures, neonatal rats of postnatal day 3-4 were used. Rats were decapitated, the spinal cords excised and roots and meninges removed in dissection buffer (HBSS, 3.4 mM NaHCO_3_, 10 mM HEPES, 33.3 mM D-glucose, 5.8 mM MgSO_4_, 0.03% bovine serum albumin (BSA), 1% pen/strep).

The lumbar part of the spinal cord was cut into 350 μm transverse sections with a McIlwain tissue chopper (Mickle Laboratory Engineering, Gomshall, UK). About 10 usable sections could be obtained from one lumbar spinal cord part. Three slices were cultured on one Millicell membrane insert (Millipore, Billerica, USA) placed in 6-well plates. Each well contained 1 ml of medium composed of 50% MEM, 25% HBSS, 25% FCS, 33.3 mM D-glucose, 1% pen/strep and 100 ng/ml GDNF to keep the motor neurons in the culture alive.

To enhance the motor neuronal survival and to guide sprouting neurites, a co-culture of the spinal cord slice with a peripheral nerve graft as a reconstructed ventral root was chosen.

As peripheral nerve graft, pieces of ulnar and median nerves were harvested from the same animals and one graft was opposed to the ventral surface of each spinal cord slice.

To check the contribution of MNPs in the tissue slices, 100 μg/ml MNPs were added to the medium for the whole culture time. Co-cultures were incubated at 37°C in humidified 6% CO_2 _atmosphere. The medium was changed 24 h after preparation and afterwards every second day.

### Immunocytochemistry of cell cultures

#### Staining procedure

After 24 h MNP incubation, cells were washed once with PBS to remove free-floating MNPs and fixed for 30 min with 4% paraformaldehyde (PFA). Fixing was followed by three times washing with PBS. Unspecific binding sides were blocked for 1 h with 10% FCS, 0.3% Triton-X in PBS. Subsequently, cells were incubated with the primary antibody overnight at 4°C. Cell type specific primary antibodies were chosen: primary neurons were stained with mouse monoclonal anti-microtubule associated protein-2 (MAP2, 1:1000, Sternberger Monoclonals, Baltimore, USA), PC12 cells with rabbit polyclonal anti-ß-III-tubulin (1:1000, Covance, Princeton, USA), microglial cells with mouse monoclonal anti-CD11b/c (1:400, BD Pharmigen, Franklin Lakes, USA) or with rabbit polyclonal anti- ionized calcium binding adaptor molecule 1 (IBA-1, 1:1000, Wako Pure Chemicals Industries, Osaka, Japan), astroglia with rabbit polyclonal anti- glial fibrillary acidic protein (GFAP, 1:500, Progen, Heidelberg, Germany) and oligodendroglia with mouse monoclonal anti-galactocerebroside (1:250, Chemicon, Billerica, USA). Schwann cells were stained with rabbit polyclonal anti-S100 (1:200, DAKO, Glostrup, Denmark) and fibroblasts with mouse monoclonal anti-fibronectin (1:200, Abcam, Cambridge, UK) antibody. Primary antibodies were diluted in PBS containing 0.3% Triton-X and 1% FCS.

After incubation overnight, cells were washed three times with PBS and incubated for 3 h with an anti-rabbit or anti-mouse Alexa Fluor 546 Dye secondary antibody (Invitrogen, Carlsbad, USA) diluted 1:200 in PBS. Cells were washed again three times with PBS and cell nuclei were counterstained with 4', 6-diamidino-2-phenylindole (DAPI, 1 μg/ml diluted in PBS, Roche Applied Science, Indianapolis, USA). After washing again with PBS, coverslips were embedded with Immu-Mount (Thermo Scientific, Waltham, USA).

#### Quantification

To quantify the cellular uptake cell type specific antibodies were used to stain cell lines followed by counterstaining all nuclei with DAPI. Control coverslips (4-5) and 10 coverslips of the MNP-incubated cells were used and 4-5 random images per coverslip (each corner and middle) were taken. Images were acquired with an AxioImager microscope (Zeiss, Jena, Germany). Total cell number (given by the DAPI-staining), the number of cell type specific stained cells (for example neurons) and the number of cells which were co-localized with the green fluorescent MNPs were counted with the AxioVision Rel. 4.8 Imaging software by Zeiss. Values were calculated as a percentage to the total cell number providing the percentage specific cell types in the cultures. Total uptake was calculated as percentage of specific cell type with MNP co-localization to the total number of this specific cell type.

In PC12 cells, the total cell number, the number of clearly differentiated cells (cells with ß-III-tubulin expression and neurite growth) and the number of differentiated cells co-localized with MNPs was quantified and calculated in a similar manner.

Statistical analysis was performed with Graph Pad Prism 4 (GraphPad Software, La Jolla, USA) using an unpaired t-test for the comparison of control and MNP group. For each cell type n = 20 images from 4-5 coverslips were chosen. For the comparison of the MNP uptake between the different cell types in the cerebellar cultures a one-way ANOVA followed by a Bonferroni post-hoc test was performed (n = 40 images from 10 coverslips). The comparison of the MNP uptake between primary neurons/PC12 cells and between Schwann cells/fibroblasts was done with an unpaired t-test (both n = 40 images from 10 coverslips). In all statistical tests a p-value ≤ 0.05 was considered to be statistically significant.

### Immunohistochemistry of organotypic co-culture

#### Staining procedure

Cultures were fixed after one week by replacing the medium with 4% PFA overnight. For immunohistochemistry, the membranes of inserts were separated from the carrier and cultures attached on membranes were stained free-floating according to the protocol described above. As primary antibodies mouse monoclonal anti-pan-neuronal neurofilament marker (1:1000, Sternberger Monoclonals), rabbit polyclonal anti-ß-III-tubulin (1:1000, Covance), mouse monoclonal anti-CD11b/c (1:400, BD Pharmigen), rabbit polyclonal anti-GFAP (1:500, Progen), rabbit polyclonal anti-S100 (1:200, DAKO) and rabbit polyclonal anti-myelin basic protein (1:200, Chemicon) were used to visualize the main cell types.

#### Imaging

**Figure 1:**
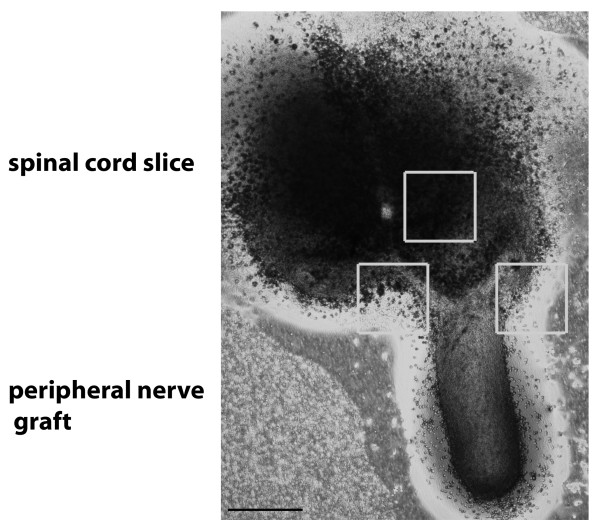
**Phase contrast of an organotypic co-culture of spinal cord and peripheral nerve graft**. Boxes indicate imaged regions for confocal microscopic analysis of different cell types including neurons, microglia, astroglia, oligodendroglia and Schwann cells. Bar represents 500 μm.

Organotypic co-cultures were imaged with a TCS SPE DMI4000 confocal microscope by Leica (Wetzlar, Germany) and edited in the Leica Application Suite 2.3. Figure 1 shows a phase contrast image of a spinal cord co-culture. This picture was taken with a cell culture microscope DMI3000 by Leica and was edited with Photoshop CS4 (Adobe Incorporated Systems, San Jose, USA) to create a collage of the whole co-culture. Images of motor neurons were taken at the ventral part of the spinal cord as marked in Figure 1 by the upper white box. Images of other cell types were taken in the vicinity of the intersection of the spinal cord slice and the peripheral nerve graft, marked by the other boxes in Figure 1.

### Electron microscopy

Cultures were fixed for 24 h with 4% PFA. They were washed with 0.1 M cacodylate buffer and incubated for 1 h with 1% osmium tetroxide, washed again with 0.1 M cacodylate buffer and 50% ethanol and incubated with 2% uranyl acetate for 1 h. Subsequently, cells were dehydrated in an ascending ethanol series and embedded in Durcupan™ ACM Fluka (Sigma, St. Louis, USA). After hardening at 68°C, cell culture plastic was removed and cells were cut in ultra-thin slices of 70 nm. Images were taken with an EM 900 transmission electronic microscope by Zeiss and edited with Adobe Photoshop CS4.

### Viability assay of cell cultures

To reveal changes in cell viability of primary cell cultures incubated with MNPs, an MTS (3-(4.5-dimethylthiazol-2-yl)-5-(3-carboxymethoxyphenyl)-2-(4-sulfophenyl)-2H-tetrazolium) proliferation assay (CellTiter 96 Aqueous one solution cell proliferation assay by Promega, Madison, USA) was used. To avoid previously reported and discussed interferences of nanoparticles with colorimetric assays and absorbance measurement [29], the experimental protocol of the MTS assay was slightly changed. Cells of both primary cell cultures were incubated on DIV 6 for 24 h with MNP medium containing different concentrations of MNPs (10, 50 and 100 μg/ml, each with n = 10 for Schwann cell/fibroblast cultures and n = 12 for cerebellar cultures). Control cells received MNP-free medium. After 24 h of incubation, the medium was removed and cells were washed once with sterile PBS. New MNP-free medium and MTS reagent were added. Cells were incubated for 3 h at 37°C in humidified 6% CO_2 _atmosphere. All values were normalized by the mean of the blank.

After incubation the absorbance was measured with a Tecan M200 microplate reader (Tecan, Männedorf, Switzerland) at 490 nm. Two values per Petri dish were determined and the mean used for statistical analysis. Statistical analysis was performed with GraphPad Prism 4 using a one-way ANOVA followed by Dunnett's post-hoc test. A p-value ≤ 0.05 was considered to be statistically significant.

**Figure 2:**
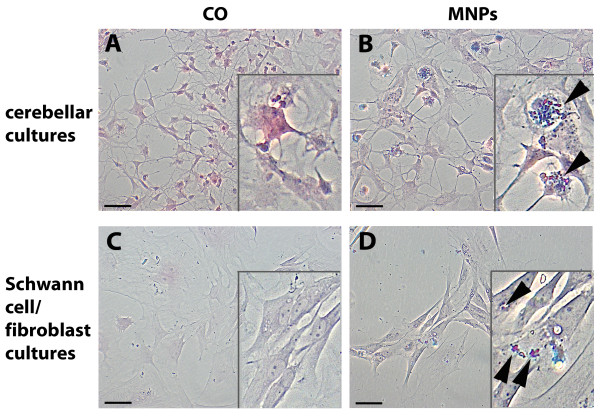
**Cellular visualization of MNPs**. Primary cerebellar and Schwann cell/fibroblast cultures are stained with nuclear fast red-aluminium sulphate (visualizing the cells) and Prussian blue (visualizing MNPs). (A) shows control (CO) cerebellar cells and (B) cerebellar cells incubated with MNPs. (C) illustrates Schwann cell/fibroblast cultures of the control and (D) incubated with MNPs. Arrows indicate stained MNPs. Bars represent 50 μm.

To check for integrity of the cell cultures, staining of cells with nuclear fast red-aluminium sulphate and Prussian blue was performed. Prussian blue stained iron-(III) oxide and visualized remaining MNPs (Figure 2). Images for each group were acquired with an AxioImager microscope (Zeiss).

## Results

### Cellular uptake

**Figure 3:**
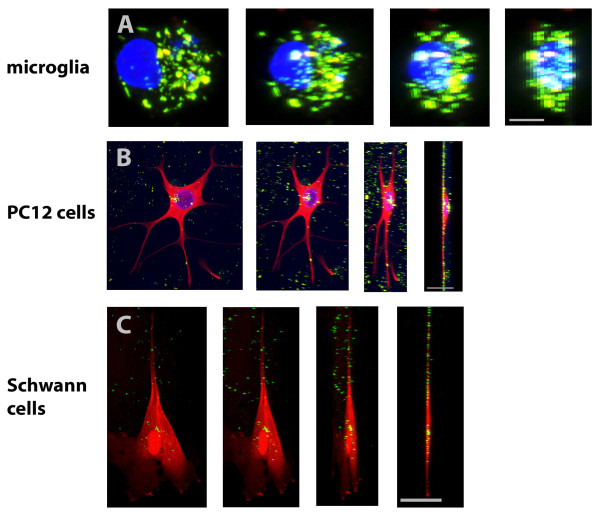
**Representative confocal 3-D-projections of cells**. Representative confocal 3D projections of a microglial cell in (A), PC12 cell in (B) and Schwann cell in (C) with MNP uptake. Image rotation is shown from left (frontal) to right (side view). Green fluorescent MNPs rotated in correlation to the nucleus and cell bodies. Additionally, MNPs were coplanar to the cell bodies indicating real uptake of MNPs in the cells. Bar represents 5 μm in (A) and 25 μm in (B) and (C).

**Figure 4:**
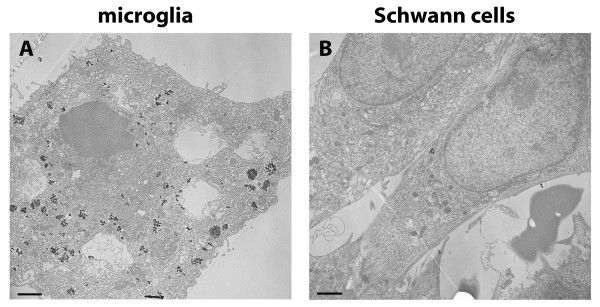
**Electron microscopy of primary cells**. Electron microscopy revealed accumulation of electronic dense MNPs in intracellular vesicular compartments, indicated by arrows. (A) shows a microglial cell of primary cerebellar cultures and (B) primary Schwann cells with uptake. Bars represent 2.5 μm.

In this study we used anionic MNPs for uptake analysis in cells of the nervous system. Previous studies reported that the uptake of anionic nanoparticles in cells is mainly mediated by endocytosis [30]. Because of this, we initially looked at the localization of MNPs using nuclear fast red-aluminium sulphate and Prussian blue staining in mixed cerebellar and Schwann cell/fibroblast cultures (Figure 2). Cultures of the control (Figure 2A, C) and the MNP group (Figure 2B, D) revealed no visual differences in culture integrity and composition. MNPs showed mainly cellular localization in the cerebellar cultures (Figure 2B, arrows) and in Schwann cell/fibroblast cultures (Figure 2D, arrows). To look in more detail, we used confocal and electron microscopy. Confocal microscopy demonstrated green fluorescent MNPs inside the cells and 3-D projections illustrated that MNPs were coplanar with the cell bodies and nucleus (Figure 3). The amount of MNPs inside cells fluctuated between the cell types; e.g. microglia (Figure 3A) displayed a high amount of MNPs whereas PC12 cells (Figure 3B) and Schwann cells (Figure 3C) took them up to a lesser extent. Electron microscopy revealed an accumulation of MNPs in intracellular vesicular compartments in microglial cells (Figure 4A, arrows) and Schwann cells (Figure 4B, arrow), confirming an endocytotic uptake mechanism.

### Cell cultures

Mixed cerebellar cultures were incubated for 24 h with MNPs on DIV 4. At this time point, mixed cerebellar cultures contained 17.7% (± 1.7% SD) detected neurons in the control cultures and 16.5% (± 2.2% SD) detected neurons in the cultures incubated with MNPs.

**Figure 5:**
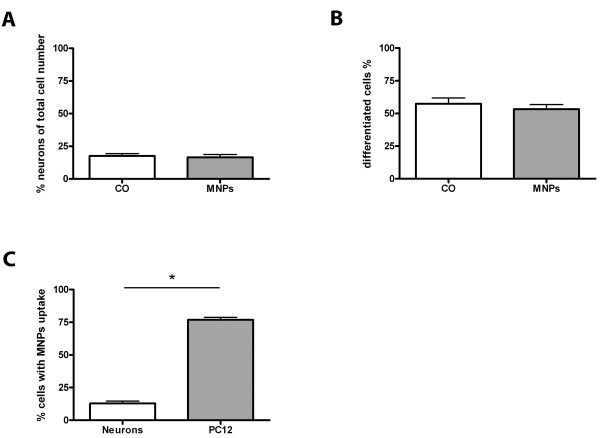
**Quantitative analysis of MNP uptake in PC12 cells and primary neurons**. All values are expressed as mean ± SD. (A) compares the percentage of primary neurons in cerebellar cultures and (B) the percentage of differentiated PC12 cells of control cells and cells after MNP incubation. MNP incubation induced no changes in the number of neurons and PC12 cells. (C) compares the number of both cell types which took up MNPs. Significantly more PC12 cells took up MNPs than primary neurons.

Cultures of PC12 cells revealed 57.5% (± 4.4% SD) clearly differentiated cells in the control and 53.4% (± 3.5% SD) differentiated cells in the cultures incubated with the MNPs. Overall, incubation of primary neurons and PC12 cells with MNPs for 24 h did not effect the cell number of both cell types (primary neurons p = 0.69, n = 20, Figure 5A; unpaired t-test, PC12 cells p = 0.47, n = 20, Figure 5B).

**Figure 6:**
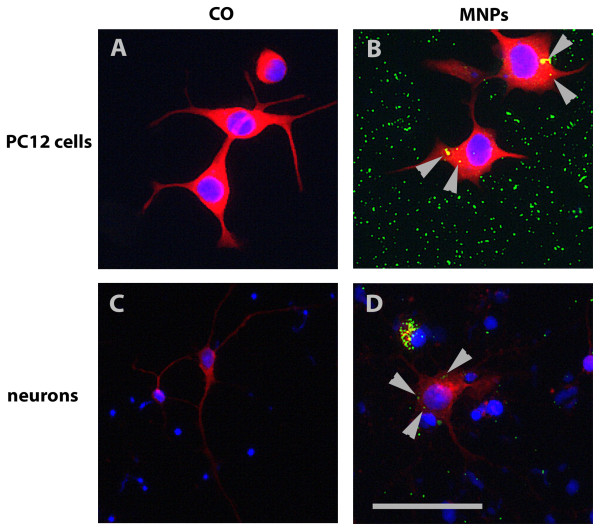
**Representative fluorescent images of PC12 cells and primary neurons**. PC12 cells in (A) and (B) were stained with an anti-ß-III-tubulin antibody (in red). (A) shows control cells and (B) cells incubated with MNPs. Primary neurons in (C) and (D) were stained with an anti-MAP2 antibody (in red). (C) demonstrates control cells, (D) cells incubated with MNPs. MNPs are fluorescent green and marked with arrows. Bar represents 50 μm.

The comparison of the uptake potential of neuronal differentiated PC12 cells with primary neuronal cells demonstrated that 76.9% (± 11.8% SD) of MNPs had been uptaken in PC12 cells compared with only 12.8% (± 13.2% SD) in the primary neurons (Figure 5C). Representative fluorescent images of PC12 cells and neurons with MNP uptake are shown in Figure 6. Control cells (PC12 cells in Figure 6A, primary neurons in Figure 6C) illustrated no green fluorescence whereas cells incubated with MNPs showed green fluorescent MNPs co-localized with PC12 cells (Figure 6B) and primary neurons (Figure 6D).

**Figure 7:**
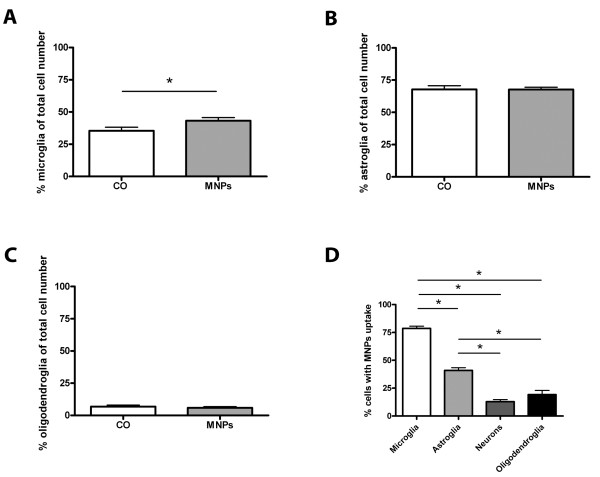
**Quantitative analysis of mixed cerebellar cultures after 24 h incubation with 50 μg/ml MNPs**. All values are expressed as mean ± SD. (A) shows the number of microglia of control cultures and cells incubated with MNPs after 24 h. The percentage of microglia increased significant with MNP incubation. The number of astroglia, shown in (B), was not influenced by MNPs. No significant differences between both groups were found for oligodendroglia in (C). In (D), a comparison of the uptake for all cell types is shown. The number of cells which took up MNPs was highest for microglia, followed by astroglia, oligodendroglia and neurons.

**Figure 8:**
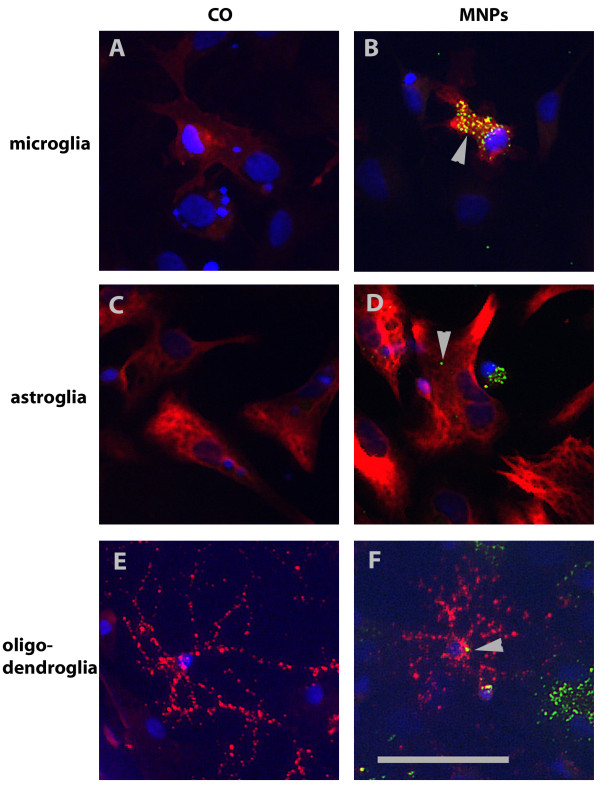
**Representative fluorescent images of glia cells in cerebellar cultures**. Cell type specific stainings are shown in red and MNPs in green (arrows). Microglia were stained with anti-CD11b/c, shown in (A) and (B), astroglia with anti-GFAP, in (C) and (D), and oligodendroglia with anti-GalC, in (E) and (F), antibodies. Glia cells in (A), (C) and (E) represent control cells, cells in (B), (D) and (F) were incubated with 50 μg/ml MNPs for 24 h. Bar represents 50 μm.

Besides neurons, cerebellar cultures contain different types of glial cells. For analysis of glia cells, we stained the cultures for glia cell type specific antibodies on DIV 7. At this time, cultures contained 35.4% (± 2.8% SD) microglia in control cultures and 43.2% (± 2.5% SD) microglia in cultures incubated with MNPs. MNP incubation for 24 h increased the number of microglia by 7.8% (± 3.8% SD) (unpaired t-test, p = 0.046, n = 20, Figure 7A). Astroglia in control cultures represented 67.8% (± 3.0% SD) and in cultures incubated with MNPs 67.7% (± 1.8% SD). No changes in the amount of astroglia were induced by MNP incubation (unpaired t-test, p = 0.98, n = 20, Figure 7B). For analysis of oligodendroglia, the cultures were stained on DIV 13 with an oligodendroglial marker. 6.9% (± 1.1% SD) oligodendroglia were found in control cultures and 5.9% (± 1.0% SD) in cultures incubated with MNPs. The percentage of oligodendroglia did not change by MNP incubation (unpaired t-test, p = 0.52, n = 19, Figure 7C). The comparison between the different cerebellar cell types revealed significant differences in the number of cells which took up MNPs (Figure 7D): 78.7% (± 12.2% SD) of microglia, 41.0% (± 14.9% SD) of astroglia, 19.2% (± 23.9% SD) of oligodendroglia and 12.8% (± 11.8% SD) of neurons showed uptake of MNPs (one-way ANOVA, p < 0.001). Representative images of glial cell types with MNP uptake are shown in Figure 8. Microglia of control cultures are illustrated in Figure 8A, astroglia in Figure 8C and oligodendroglia in Figure 8E. All lacked green fluorescent signals. The green fluorescent MNPs are clearly seen in cultures incubated with MNPs for 24 h (see microglia in Figure 8B, astroglia in Figure 8D and oligodendroglia in Figure 8F, arrows). The images also show differences in the cellular uptake of MNPs. Stained microglia clearly demonstrated a higher content of MNPs per cell than the other cell types (Figure 8B, arrow).

Although there is an increase in cell number of microglia in cultures incubated with MNPs, the viability assay revealed no significant differences between control cells and cells incubated with 10, 50 and 100 μg/ml MNPs (data not shown) (one-way ANOVA, p > 0.05, n = 12).

**Figure 9:**
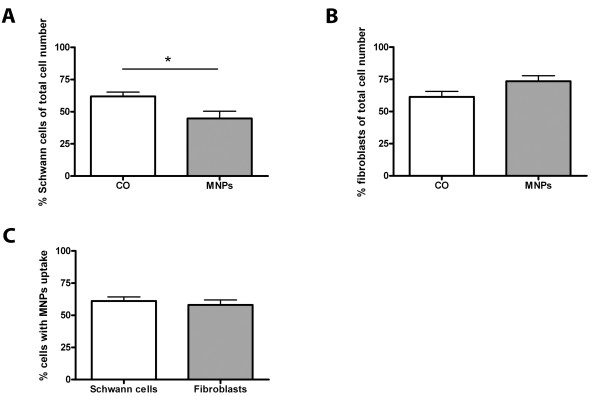
**Quantitative analysis of primary Schwann cell/fibroblast cultures after 24 h incubation with 50 μg/ml MNPs**. All values are expressed as mean ± SD. In (A), the number of Schwann cells is compared between cells of the control and cells incubated with MNPs. MNP incubation decreased the percentage of Schwann cells. The comparison of control cells and cells incubated with MNPs for fibroblasts is shown in (B). The number of fibroblasts in the cultures was not influenced by MNP incubation. (C) shows the comparison of the uptake for both cell types. The number of cells which took up MNPs was similar in both cell types.

**Figure 10:**
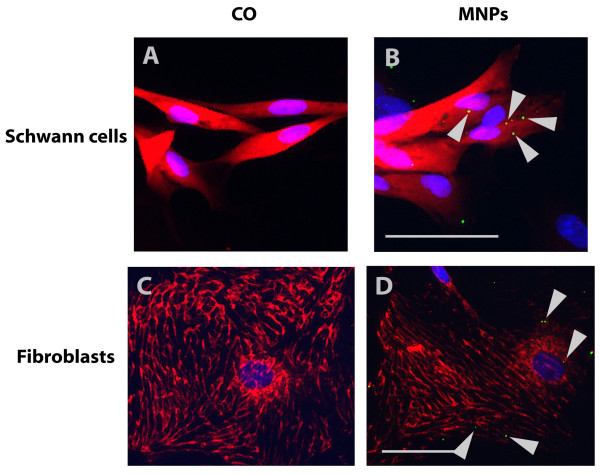
**Representative fluorescent images of all cell types of primary mixed Schwann cell/fibroblast cultures**. Cell type specific stainings are shown in red and MNPs in green (arrows). Schwann cells in (A) and (B) are stained with anti-S100 antibody and fibroblasts in (C) and (D) with anti-fibronectin antibody. Images (A) and (C) illustrate control cells without MNPs and images (B) and (D) cells after MNP incubation. Bars represent 50 μm.

Mixed Schwann cell/fibroblast cultures were used on DIV 7 and contained 61.9% (± 3.3% SD) Schwann cells in control cultures. The incubation with MNPs induced a decrease of 17.1% (± 5.5% SD) in the number of Schwann cells to 44.8% (± 5.5% SD) Schwann cells (unpaired t-test, p = 0.012, n = 20, Figure 9A). 61.4% (± 4.4% SD) of fibroblast were found in the control cultures and 73.5% (± 4.3% SD) in cultures incubated with MNPs indicating a trend of an increased number of fibroblasts (unpaired t-test, p = 0.054, n = 20) compared to controls (Figure 9B). Nevertheless, Schwann cells seem to be more sensitive to the MNPs then fibroblasts, but their uptake capacity is similar: 61.1% (± 3.2% SD) of Schwann cells and 58.1% (± 3.9% SD) of fibroblasts showed uptake of MNPs after 24 h incubation time (unpaired t-test, p = 0.544, n = 40, Figure 9C). Representative images of Schwann cells and fibroblasts with MNP uptake are illustrated in Figure 10. Schwann cells and fibroblasts of the control cultures lacked green fluorescent signals (Figure 10A, C), but cells incubated with MNPs showed green fluorescent MNPs co-localized with the cells (see Schwann cells in Figure 10B and fibroblasts in Figure 10D, arrows).

The viability assay revealed no significant differences in viability between control cells and cells incubated with 10, 50 or 100 μg/ml MNPs for 24 h (data not shown) (one-way ANOVA, p > 0.05, n = 10).

### Organotypic co-cultures

**Figure 11:**
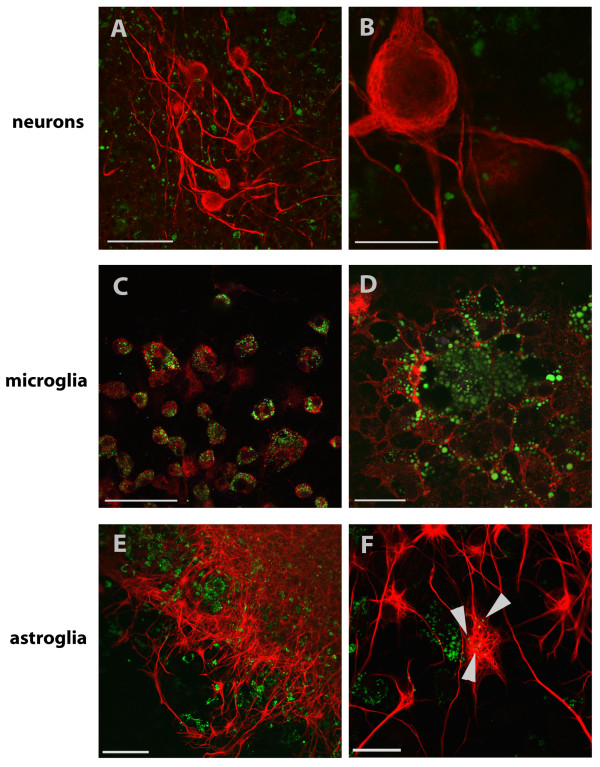
**Immunohistochemical staining of organotypic spinal cord co-cultured with peripheral nerve graft**. Co-cultures were incubated for one week with 100 μg/ml green fluorescent MNPs and were stained with anti-pan-neuronal neurofilament to visualize neurons, shown in images (A) and (B). Neurons do not display co-localization with green fluorescent MNPs. Microglia in (C) and (D) were visualized with the microglial marker anti-CD11b/c and demonstrated high amount of MNPs localized in vesicles. Astroglial cells in (E) and (F) were stained with anti-GFAP antibody and showed moderate MNP co localization (arrows). Bars in (A) and (E) represent 75 μm, in (C) 50 μm and in (B), (D) and (F) 25 μm.

Organotypic co-cultures of spinal cord and peripheral nerves were used to verify the results of the dispersed cultures of the CNS and PNS in a more complex model. MNPs were not found inside of motor neurons and their neurites (Figure 11A, B) or in Schwann cells guiding sprouting neurites. Microglia revealed vesicular accumulation of huge amounts of MNPs inside the cells (Figure 11C, D). Astroglia (Figure 11E, F) and oligodendroglia took up MNPs and showed single accumulation. Thus, uptake in organotypic culture resembles the uptake observed in dispersed cultures.

## Discussion

Literature regarding primary cell cultures, especially of the nervous system and nanoparticles, is limited. Most previous studies in nanosciences use cell lines such as PC12 as a neuronal model [16,18]. However, cell lines are often tumour cells or immortalised cells behaving differently than primary cells [21,22]. PC12 cells show a reliable uptake of nanoparticles: Non-functionalized polymer-coated nanoparticles were found to be internalized in the cytoplasm in a highly non-specific manner [31], without toxic effects. Pisanic II et al. [18] demonstrated a qualitative proportional uptake of anionic nanoparticles, indicating non-limited uptake for the used concentrations. Even biological functions of PC12 cells could be influenced by nanoparticles. Hussain et al. [32] for example, depleted dopamine and dopamine metabolites with manganese oxide particles in a dose-dependent manner. Comparing differentiated PC12 cells and primary cerebellar neurons in our study, we found significant differences in the uptake of MNPs. A high cellular uptake was observed in the PC12 cells compared with only about 13% in primary neurons. Obviously, PC12 cells are a questionable choice for exploring the response of neurons to nanoparticles and do not predict neuronal uptake of primary neurons.

We used mixed cerebellar cultures to model interactions of the major cell types of the CNS. They contain, apart from neurons, microglia, astroglia and oligodendroglia, which all have the potential to internalize MNPs [33-35]. However, the uptake of MNPs was distinct between the different cell types of these primary cultures. MNPs were found to be predominantly taken up by microglia.

Microglial cells are resident macrophages of the CNS, sensing cell damage and pathogens [36]. If microglia are activated by a stimulus, they change their morphology, up-regulate immunologically relevant molecules such as chemokine receptors, major histocompatibility complex molecules and phagocytic receptors [37,38], and phagocytose possible pathogens. Lundqvist et al. [39] showed that nanoparticles become surrounded by a protein corona if they are suspended in blood plasma. Different particle sizes and charges resulted in different coronas which were composed for example of immunglobulins, lipoproteins and complement factors. This protein corona results in an opsonization of nanoparticles and phagocytosis by macrophages and microglia [40,41]. Given that the MNPs used in this study were suspended in serum containing medium, it is possible to assume an opsonization of the MNPs by serum proteins which stimulate the microglia to phagocytose. We found that nearly 80% of the microglia in these cultures took up MNPs. Additionally, microglia proliferate in response to pathogens [42,43]. This was demonstrated in our study, too. The number of microglia increased after incubation with MNPs. This indicates activation of the microglia through the MNPs and suggests a problem for *in vivo *application. Microglia would phagocytose the administered MNPs before they could target other cells such as neurons. Additionally, they would trigger immune responses and influence other cell types. This could counteract with the goal to use the MNPs for the promotion of nerve regeneration in pathological conditions.

MNPs were also internalized by astroglia and oligodendroglia, but to a lesser extent. Phagocytosis is described in the literature for both glia types, too. However, the potential seems to be lower than for microglia and differs in velocity between the cell types [44-47], which could explain the different uptake of the glial cell types found in our study. In purified cultures, astroglia as well as oligodendroglial precursor cells revealed a satisfying uptake of different kinds of nanoparticles [33-35]. Purified cultures just give information about single cell types and lack the interactions between the various cell populations that can be found in tissues. Also Pickard and Chari [33], who showed robust nanoparticle uptake in purified primary microglia, pointed out the importance of studies comparing and analyzing nanoparticle uptake in the major cell populations of the CNS. Our study, using mixed cultures of the CNS and PNS, bridges this gap and take the interactions of the cell types into account. Additionally, Pickard and Chari [33] predicted in their study "... that the rapid and extensive MNP uptake by endogenous microglia could represent a significant 'extracellular' barrier to particle uptake by other neural cell subpopulations." The results of our study confirm their prediction. As mentioned above, most MNPs were phagocytosed by the microglial cells in the mixed cultures which presumably reduce the amount of MNPs for uptake by other cells.

The mixed Schwann cell/fibroblast cultures model the PNS environment. The PNS environment and especially the Schwann cells influence nerve regeneration in the nervous system, too. Both cell types took up MNPs and do not show phagocytosis under normal conditions. But both cell types are able to activate phagocytic potential with appropriate stimulation. Schwann cells are able to activate phagocytic functions during injury of the nerve and clear the injury site of cell and myelin debris [14,48]. Also, *in vitro*, Schwann cells retain this function and can be stimulated to phagocytose applied myelin membranes within 1 h [49]. Fibroblasts, on the other hand, reveal substantial phagocytosis in wounds as well as under *in vitro *conditions [50-52]. In purified cultures, both cell types revealed reliable uptake of different kinds of nanoparticles [53,54].

Besides phagocytosis, different types of endocytotic pathways or diffusion [48] of the negatively charged MNPs, facilitated by positively charged membrane domains [55], could also be possible mechanisms for the cellular uptake of MNPs in astroglia, oligodendroglia, Schwann cells and fibroblasts and might explain the particular MNP uptake of the different cell types in our experiments.

Dispersed cultures do not model the *in vivo *situation and environment in total. Cells grow in a monolayer and lack certain cell-cell connections. Organotypic cultures offer a more complex and tissue-like environment for the cells. Due of this factor, they model the *in vivo *environment more adequately than dispersed cultures do. We compared our results of the dispersed primary cell cultures of the CNS and PNS to the organotypic co-culture of spinal cord slices (CNS) and peripheral nerve graft (PNS). The uptake of MNPs was similar between both systems. MNPs were internalized predominately in microglia of the spinal cord slice, but not in neurons. Astroglia, oligodendroglia and Schwann cells revealed uptake too, but again to a lesser extent.

Cellular uptake of exogenous particles might induce toxic effects. Toxicity of nanoparticles is a serious problem for biological and medical purposes. However, nanoparticles are not basically toxic. In human dermal fibroblasts, Gupta and Gupta [56] compared non-coated superparamagnetic iron oxide nanoparticles with polymer-coated particles and revealed a toxic effect of the non-coated particles and no toxic effect of the coated ones. On the other hand, Karlsson et al. [57] compared different metal oxide nanoparticles of different sizes concerning their influences on viability and mitochondrial and DNA damage. They found size- and metal oxide-dependent toxic effects and reported low toxicity of iron oxide particles. The MNPs used in this study showed only low toxicity towards Schwann cells. This would make them useful candidates for *in vivo *applications in the nervous system concerning toxicity at first view.

Given that dissociated and organotypic cultures show similar uptake results, it is likely that these commercial MNPs, although they show low toxicity, will not be easy to use *in vivo *in the nervous system. They activate the immune cells of the CNS and are phagocytosed by microglia in high numbers. Thus, for targeting cells other than microglia, the MNPs have to be tagged with a cell-type specific marker. Additional functionalizing of MNPs with receptor agonists for a receptor-mediated uptake mechanism [58] or a more lipophilic coating to increase membrane permeability can improve cell-specific uptake [59]. Once the MNPs are taken up in a sufficient amount and frequency by specific cells of the nervous system, they can be used for locally influencing cell responses to axonal injury, for example with the delivery of siRNA or transplantation of supporting cells. Also, delivery of local drugs, growth factors, hormones and extracellular matrix molecules in and on the cell surface to influence cell responses [49,60] is possible through manipulation of MNP localization with an external magnetic field.

## Conclusion

Nanoparticles offer a large range of applications in life and neurosciences. Their functionality often depends on the fact that they are internalized by cells. Thus, we analyzed the uptake of commercially available MNPs in cells of the nervous system and were able to show that PC12 cells differ in their uptake to primary neuronal cells, and thus are not an appropriate choice for answering questions concerning the response of neurons to nanoparticles. MNPs in primary cerebellar and in organotypic co-cultures were found to be predominantly taken up by microglia, suggesting a problem for *in vivo *application of these commercial MNPs because of the immune response. Future research needs to overcome this immune system activation with cell-specific targeting and additional functionalization.

## Abbreviations

ANOVA: Analysis of variance; BSA: Bovine serum albumin; CNS: Central nervous system; DAPI: 4',6-diamidino-2-phenylindole; DIV: Day in vitro; DMEM: Dulbecco's modified eagle medium; FCS: Fetal calf serum; GDNF: glial cell line-derived neurotrophic factor; GFAP: Glial fibrillary acidic protein; HBSS: Hank's balanced salt solution; HEPES: 4-(2-hydroxyethyl)-1-piperazineethanesulfonic acid; hTERT-BJ1: Infinity™ telomerase-immortalised primary human fibroblasts; HUVECs: Human umbilical vein endothelial cells; IBA-1: Ionized calcium binding adaptor molecule 1; MAP2: Microtubule associated protein-2; MEM: Eagle's minimal essential medium; MNPs: Magnetic nanoparticles; MTS: 3-(4,5-dimethylthiazol-2-yl)-5-(3-carboxymethoxyphenyl)-2-(4-sulfophenyl)-2H-tetrazolium; NGF: Nerve growth factor; PBS: Phosphate buffered saline; PC12 cells: Rat pheochromocytoma cell line; PFA: Paraformaldehyde; PNS: Peripheral nervous system; Pen/strep: Penicillin/streptomycin; RPMI: Roswell park memorial institute.

## Competing interests

The authors declare that they have no competing interests.

## Authors' contributions

JP performed all cellular work, statistical analysis and microscopic work. She largely wrote the manuscript. PC and GFG performed all physical measurements of the magnetic nanoparticles and their analysis. They contributed to the writing of the manuscript. HF contributed the writing of the manuscript, too. GK conceived the experiments and contributed largely to the final version of the manuscript. All authors read and approved the final manuscript.
